# Deficits in Conditional Discrimination Learning in Children with ADHD Are Independent of Delay Aversion and Working Memory

**DOI:** 10.3390/jcm8091381

**Published:** 2019-09-03

**Authors:** Hasse De Meyer, Tom Beckers, Gail Tripp, Saskia van der Oord

**Affiliations:** 1 Research Unit Behavior, Health and Psychopathology, KU Leuven, Tiensestraat 102, 3000 Leuven, Belgium; hasse.demeyer@kuleuven.be (H.D.M.); Tom.Beckers@kuleuven.be (T.B.); 2 Department of Psychology, Faculty of Behavioural Sciences, HELP University Subang 2, Persiaran Cakerawala, Seksyen U4, Shah Alam 40150, Selangor, Malaysia; 3 Leuven Brain Institute, KU Leuven, 3000 Leuven, Belgium; 4 Human Developmental Neurobiology Unit, Okinawa Institute of Science and Technology Graduate University, 1919-1 Tancha, Onna, Kunigami District, Okinawa, Prefecture 904-0412, Japan; Tripp@oist.jp; 5 Developmental Psychology, University of Amsterdam, Nieuwe Achtergracht 129, 1018 WS Amsterdam, The Netherlands

**Keywords:** attention-deficit/hyperactivity disorder (ADHD), conditional discrimination learning, diagnosis, arbitrary delayed matching-to-sample, memory, delay aversion, learning

## Abstract

Adaptive behavior requires the adjustment of one’s behavioral repertoire to situational demands. The learning of situationally appropriate choice behavior can be operationalized as a task of Conditional Discrimination Learning (CDL). CDL requires the acquisition of hierarchical reinforcement relations, which may pose a particular challenge for children with Attention Deficit Hyperactivity Disorder (ADHD), particularly in light of documented deficits in short-term/working memory and delay aversion in ADHD. Using an arbitrary Delayed Matching-To-Sample task, we investigated whether children with ADHD (N = 46), relative to Typically Developing children (TD, N = 55), show a deficit in CDL under different choice delays (0, 8, and 16 s) and whether these differences are mediated by short-term/working memory capacity and/or delay aversion. Children with ADHD demonstrated poorer CDL than TD children under 8 and 16-second delays. Non-delayed CDL performance did not differ between groups. CDL differences were not mediated by short-term/working memory performance or delay aversion. Moreover, CDL performance under an 8-second delay was a better predictor of clinical status than short-term/working memory performance or delay aversion. CDL, under conditions of delay, is impaired in children with ADHD. This may lead to difficulties discriminating between different situational demands and adapting behavior according to the prevailing reward contingencies or expectations.

## 1. Introduction

In daily life, we are constantly confronted with situations that require us to make choices that carry consequences. A child may choose to do a chore that is boring but will yield praise eventually, or go play and have fun at the risk of later getting scolded. It is generally accepted that this real-life choice behavior is driven by the learned consequences that follow our choices. In reality, however, the consequences of our choices are often context-specific (e.g., depending on the tone and mood of a parent, a child prioritizing immediate pleasure at the cost of disobeying instructions may find themselves maximizing reinforcement or not) [1]. Adjusting our behavior to the situation to maximize reward requires that we identify the situation and integrate information about the consequences of our choices [2]. This ability is essential for the emergence of adaptive behavior and enables us to meet the social, emotional, and cognitive demands of our environment [2].

While clinically, children with Attention Deficit Hyperactivity Disorder (ADHD) often appear to struggle with behavioral adjustment to changing reinforcement contingencies [3] and to make impulsive choices without taking account of the consequences of their actions [4], empirical evidence for a basic deficit in instrumental learning in children with ADHD is limited [5,6]. Some previous studies report differences in instrumental learning between children with ADHD and typically developing (TD) children [7,8,9]. However, these differences may result from the use of complex tasks that involve the application of cognitive strategies [10] in addition to instrumental learning [5]. Nigg and Casey have suggested that children with ADHD, in particular, may fail to take account of their current situation and the accompanying expectations, and thus fail to act per environmental expectations [4]. This hypothesis has yet to be experimentally tested. The capacity to adjust choice behavior to situational demands or reinforcement contingencies can be modeled in the laboratory as a Conditional Discrimination Learning (CDL) task. A CDL task involves a specific form of instrumental learning [1,11]. In a CDL task, which of multiple possible choice responses (e.g., selecting an arbitrary symbol from an array of options) is followed by reinforcement on a given trial, is dependent on the context (e.g., the presentation of another arbitrary symbol at the start of the trial) [1,12]. Despite its obvious relevance for behavioral adjustment, the CDL ability of children with and without ADHD has received very little attention [13,14].

ADHD is often seen as involving the neurodevelopmental dysregulation of processes that control cognitive functions [15]. This leads to a general failure to adapt feelings, thoughts, and actions to the expected social norms, prompting impairment in daily life [15,16]. One of the mediators in this cognitive-behavioral process is a higher-order, top-down control system, denoted as the Executive Functioning (EF) system [4,17,18]. The EF system consists of several dissociable but overlapping functions (e.g., inhibition, working memory, planning, cognitive flexibility). Frequently reported EF impairments in ADHD include working memory deficits (especially spatial working memory but also short-term memory) and impairments in inhibition and set-shifting [19]. Working memory, or the capacity to hold information online while manipulating it [19,20], shows the largest differences (with a moderate effect size) between children with ADHD and TD children [19,21,22]. In daily life, working memory is important for controlling behavior and guiding decision-making [23].

Motivational deficits are also thought to contribute to ADHD symptomatology [15,24,25,26]. Children with ADHD are particularly prone to delay aversion, presumably due to disruptions in the reward system [27]. Behaviorally, children with ADHD attempt to escape delays as much as possible, preferring, for instance, a small immediate over a larger delayed reward. When a delay cannot be escaped, the delay may result in impulsive, inattentive, or hyperactive behavior aimed at reducing the individual’s experience of delay, i.e., experiential avoidance [27,28].

As with working memory, delay aversion cannot account for all symptoms of ADHD; the effect size for differences between children with ADHD and TD children on delay aversion tasks is generally small [29]. Like working memory deficits, enhanced delay aversion is thought to be one of the multiple pathways potentially involved in the pathology of ADHD [29]. Dopaminergic signaling is purportedly involved in deficits in EF and delay aversion in ADHD, but dopamine signaling is also critically involved in associative learning/CDL [2,30]. Conditional discrimination learning, i.e., learning to select the correct or most adaptive behavioral option in light of contextual information may be especially affected when a delay occurs between the presentation of contextual information and availability of behavioral options. In bridging this delay, memory components, often impaired in ADHD, such as short-term memory or working memory, might mediate performance. Additionally, choice delay and the associated delay of reinforcement might trigger a negative emotional state, i.e., delay aversion. Of note, in real-life situations, a delay between contextual information (e.g., a teacher announcing the topic of a class) and the execution of choice behavior (e.g., a pupil picking the appropriate book from their bag) is the rule rather than the exception.

An established protocol to assess CDL in clinical and non-clinical samples is the arbitrary Matching-To-Sample task (aMTS) [12,31]. In a non-arbitrary MTS task, participants are required to match the sample to the identical choice stimulus. In an aMTS task, a participant is presented with a choice environment with an arbitrary sample stimulus (e.g., S_1_ or S_2_) together with different choice stimuli (e.g., C_1_ and C_2_). Which choice is correct, is conditional on the sample stimulus presented; e.g., when S_1_ is presented, the correct response is C_1_, and when S_2_ is presented, the correct response is C_2_. The sample stimuli and choice stimuli bear no intrinsic relationship; the sample-to-choice associations need to be learned [32]. In an arbitrary *Delayed* Matching-To-Sample task (aDMTS), a time interval is moreover inserted between the offset of the sample stimulus and the onset of the choice stimuli. Although to the best of our knowledge not experimentally tested, some researchers attribute delay-related performance impairments to short-term memory [33,34], whereas others claim that sample-choice delays burden working memory [35,36]. Besides the possible load on short-term and/or working memory implied by an aDMTS task, the task also involves the need to tolerate a delay, as participants are forced to wait before they make a choice and receive feedback. This might create a negative affective state (delay aversion) that could interfere with learning the correct sample-choice associations. Previous research in children with ADHD has shown that tolerating as little as a 6-s delay (and especially a 14-s delay) is experienced as aversive and can be associated with hypoactivation of “emotional” brain regions [27].

As far as we are aware, only one study has investigated CDL in children with ADHD [14]. In a modified, non-delay aMTS task, Gitten et al. [14] found that the performance of children with ADHD did not differ from TD children. However, as only 12 children with ADHD participated and given the heterogeneity of ADHD, the study might have been underpowered to detect differences. The current study aimed to more comprehensively assess CDL in children with ADHD, under different delays, while also assessing the contribution of memory (short-term/working) and delay aversion to task performance.

First, based on behavioral manifestations of ADHD, we hypothesized that a CDL deficit might be involved. When limited memory load was involved (no sample-choice stimuli delay), we predicted that children with ADHD would exhibit a deficit in CDL, compared to TD children, and would thus perform more poorly on an aMTS task [12,37]. When memory load was increased in the aDMTS task (with delays of 8 or 16 s imposed between sample and choice stimuli), we expected the performance difference between children with ADHD and TD children to increase based on previous reports of impaired working memory and short-term memory in this group [19]. Further, we expected that children with ADHD would exhibit difficulties with delay aversion, short-term memory, and working memory relative to TD children, and we hypothesized that both memory (short-term and/or working memory) and delay aversion mediated the relationship between group status (ADHD/TD) and CDL performance when delays were imposed. Finally, we examined the predictive value of CDL performance for classifying children as ADHD or TD.

## 2. Materials and Methods

### 2.1. Participants

Fifty-one children with an existing clinical diagnosis of ADHD volunteered for the study. ADHD diagnoses were confirmed with the Diagnostic Interview Schedule for Children, Parent Version (PDISC) [38]. Fifty-six typically developing children also participated in the study. All participants were between 8 and 12 years of age and were recruited through flyers and posters in health care institutions (ADHD) and elementary schools (TD). Inclusion criteria are reported below.

For all participants: (1) An estimated Full-Scale IQ ≥ 80, as measured by the Block Design and Vocabulary subtest, short version of the Dutch Wechsler Intelligence Scale (WISC) [39]. Results of this subtest form correlate highly (*r* = 0.86) with full-scale IQ scores and have satisfactory reliability [40]. (2) Presence of a non-(sub)clinical score (below the 95th percentile) on the Conduct Disorder scale of the Dutch translation of the parent version of the Disruptive Behavior Disorder Rating Scale (DBDRS) [41]. Participants with ADHD subsequently participated in an intervention study for which the presence of symptoms of Conduct Disorder (CD) was an exclusion criterion. The DBDRS is a 42-item questionnaire, including satisfactory measures of Inattention, Hyperactivity/Impulsivity, Oppositional Defiant Disorder (ODD), and Conduct Disorder (CD), based on DSM-IV (Diagnostic and Statistical Manual of Mental Disorders, fourth edition) criteria [41]. (3) Absence of Autism Spectrum Disorder (ASD), as well as any sensory, neurologic, or motor disorder, as reported by the parents. (4) Sufficient understanding of the Dutch language to understand the task and the instructions, and (5) absence of medication intake other than methylphenidate for the participants with ADHD; methylphenidate was withheld for 24 h before testing to assure sufficient washout [42].

For ADHD: A prior clinical diagnosis of Attention Deficit Hyperactivity Disorder according to the DSM-IV, established by a psychologist or psychiatrist and validated by the PDISC. The PDISC is a structured DSM-IV-based interview with adequate psychometric properties [38].

For TD: Scoring within the normal range (below the 90th percentile) on the ADHD subscales of the DBDRS and absence of any other psychiatric disorder as reported by the parents.

### 2.2. Conditional Discrimination Learning Task

**Figure 1:**
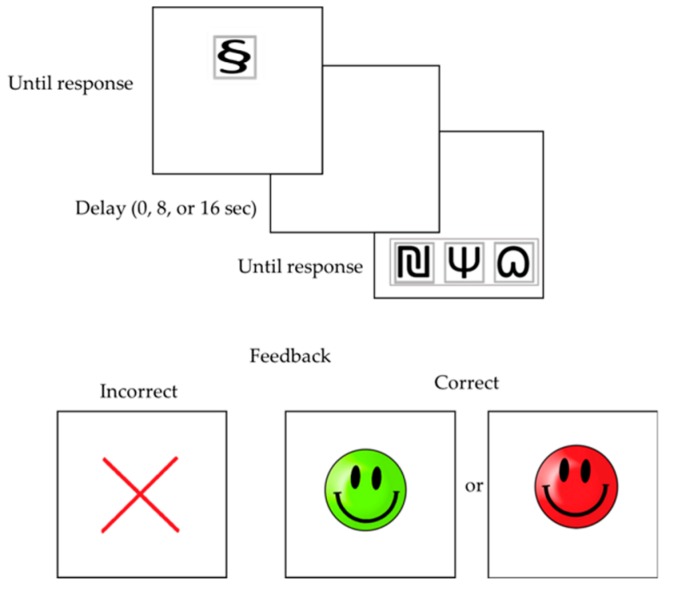
The sequence of the CDL (Conditional Discrimination Learning) task. The sample stimulus was presented together with a sound and remained on the screen until the child responded by touching the stimulus on the touch screen. During the delay (if any), a blank screen was presented. Then, the choice stimuli were presented and remained on the screen until the child responded on the touch screen. Incorrect responses were followed by a red cross, whereas correct responses were followed by a red or green smiley face (random presentation of colors). After 2 s, the next trial was presented.

Our CDL task involved a widely-used DMTS procedure [12,34,37] and was optimized for 8–12 years old children through pilot testing. In a DMTS task, on each trial, participants are presented with a sample stimulus (S_1_ or S_2_) followed by a retention interval and two choice stimuli (C_1_ & C_2_) [37,43]. Their task is to choose the stimulus identical to the sample stimulus that is presented. In its standard form with delay, the DMTS task can be considered a working memory task [36,43]. To convert it to a CDL task, rather than an identity matching task, an aMTS procedure is used [37,43]. In an aDMTS task (Figure 1), the sample stimulus and the correct choice stimulus are not related by identity but are assigned arbitrarily, such that response-outcome associations are conditional: choosing C_1_ is arbitrarily assigned to be the correct response in the presence of S_1_, whereas selecting C_2_ is the correct response for S_2_ [43]. The difficulty of an (arbitrary) DMTS task is partially determined by the length of the retention interval between presentation of the sample and choice stimuli [32,43]. Given the arbitrary features, bridging the time between the disappearance of the sample stimulus and the appearance of the choice stimuli is thought to rely on working memory [36].

Modeled on earlier research [12], the task consisted of a practice phase to familiarize participants with the task followed by a test phase. Before the task started, children were instructed to find the correct choice stimuli for the presented sample stimuli. In the practice phase, children were presented with a sample stimulus (S_1_ or S_2_) on the touch screen, accompanied by a sound to make children attend to the screen. To ensure optimal attention of the participant during each trial, the task was self-paced (the stimuli stayed on the screen until the participant responded by touching the stimuli on the screen). After pressing the sample stimulus, three choice stimuli appeared (C_1_, C_2,_ C_3_). The third choice stimulus was added as a distractor to increase task difficulty, based on a series of pilot studies with TD children of the same age as those in the current study. Pilot testing showed that with 2 sample and 2 choice stimuli, a high percentage of children reached maximum performance, whereas, with 2 sample and 4 choice stimuli, the task was too difficult for most children. To identify the relevant associations (S_1_-C_1_ and S_2_-C_2_), children were verbally guided through the practice phase [12]. For the first three pairings in the practice session, the sample and choice stimuli were identical meaningful objects (e.g., chair, flower, etc.) and children had to simply match them. In the next four trials, the stimuli were distinguishable, randomly chosen abstract figures, and there was no intrinsic relationship between the sample and choice stimuli. The appearance of which sample stimulus (S_1_ or S_2_) and the position of the three choice stimuli (C_1_, C_2_, C_3_) was determined randomly for each trial (12 options). The correct sample-choice association was determined in advance and was not counterbalanced. A correct choice was rewarded with a smiley face (red or green, randomly selected), and an incorrect response was followed by a red cross. The stimuli were presented on a white 15-inch touch screen. The sample and choice stimuli were black and white, measuring 5 cm by 5 cm, and the feedback stimuli 10 cm by 10 cm.

In the test phase, three different delays were applied between the presentation of the sample stimulus and the appearance of the choice stimuli (0 s, 8 s, and 16 s). The sample and choice stimuli were again abstract figures, similar to the ones used in the final trials of the practice phase. During the delay, a blank screen was presented. The 0-seconds delay test involved 36 trials (3 blocks of 12 trials each, while the 8-seconds and 16-seconds delays included 24 trials each (2 blocks of 12 trials). The order of the delay blocks was fixed, to minimize within-group variability, due to order effects. With every new block of trials, new stimuli were used, and new associations had to be acquired. The outcome measure of interest was *the percentage of correct trials per delay* (0, 8, or 16 s) [29], calculated across the last 20 trials of each delay (last 32 trials for delay 0), as performance on the first four trials was not indicative of learning (i.e., correctly responding on the first 4 trials could only result from chance because children did not have any opportunity to learn the correct sample/choice combinations for that block yet).

Corsi Block Tapping Task (CBTT) The digital CBTT is a computerized version of the Corsi Block Tapping Test [44], developed and made available by Dr. Sebastiaan Dovis, Alexander Chatzizacharias, M.Sc., and Marvin Soetanto, M.Sc., University of Amsterdam (for a free copy of the digital CBTT, please contact Dr. Dovis, s.dovis@uva.nl). It is used to assess non-verbal visuospatial short-term memory and working memory capacity. In the task, pictures of nine cubes (blocks) are presented on a 15-inch touch screen. A sequence of blocks lights up one by one, accompanied by a sound, and the participant needs to reproduce this sequence in the same (CBTT forward, visual-spatial short-term memory) or the reverse order (CBTT backward, visual-spatial working memory). The minimum sequence length of blocks presented was three with a maximum of nine blocks in each sequence. Each sequence length was presented three times, and no sequence was identical. When a child succeeded in reproducing at least one (out of three) sequence length in the correct order, the sequence length was increased by one block. The task ended when the child failed to complete a single sequence at a given difficulty level. The condition that was assessed first (backward or forward) was counterbalanced across participants. The outcome measure of both CBTT-forward and CBTT-backward was the total number of trials they performed correctly, with a maximum of 18, which was transformed into a percentage score.

The Maudsley Index of Delay Aversion (MIDA) is a game-based computer task developed to assess delay aversion in children [45]. During this task, children have to choose (20 times for each of the 2 conditions) between a small immediate reward (1 point after a 2-second delay) or a large delayed reward (2 points after a 30-second delay). In the no-post delay condition, children can escape the delay by choosing a small reward; however, when they want the large reward, they are required to wait 30 s. After selecting a reward (by clicking the mouse), the next trial starts. In the post-delay condition, a delay of 30 s is inserted before the next trials starts, regardless of which reward is chosen (small or large reward). The goal of the game is to earn as many points as possible. Task order was counterbalanced across participants. Only the data from the post-delay condition are used in the current study, as this condition has repeatedly been shown to distinguish between ADHD and TD children [46]. The outcome variable used in the analyses was the percentage of choices for the delayed reward (post-delay condition only). Psychometric studies have shown that the MIDA has satisfactory test-retest reliability and validity [45,46].

### 2.3. Procedure

Before participating, parents and children were informed about the study and gave their written consent to participate. While the structured interview and questionnaires were being administered to the parents, the experimental tasks were completed by the children in a quiet, distraction-free room. All participants completed the CDL task (consecutive 0, 8, 16 s delay) first, followed by either the MIDA (no-post delay or post-delay, counterbalanced) or the CBTT (forward or backward, counterbalanced) and other tasks not part of the current study. Finally, they completed the Block Design and Vocabulary subtests of the WISC-III-NL. Counterbalancing of MIDA and CBTT over participants was based on diagnosis, age, and gender, resulting in 8 different testing orders. At the end of the test session (100 min), parents received ten euros, and children received a small gift.

The study was conducted per the Declaration of Helsinki and its later amendments. The Social and Societal Ethics Committee of the Faculty Psychology and Educational Sciences at KU Leuven approved the study (G-2015 01 156).

## 3. Results

### 3.1. Demographic Characteristics

Six children (five ADHD and one TD) did not meet the inclusion criteria, and their data were excluded from the dataset (IQ < 80, N = 4; no confirmation of ADHD through a structured interview, N = 2). Out of 46 children who met DSM-IV criteria [47] for a diagnosis of ADHD (34 combined, 10 inattentive, and two hyperactive/impulsive), 52.17% of them were taking stimulant medication. The ADHD and TD groups were compared on demographic characteristics using Chi-Square for categorical and one-way ANOVA for continuous variables (see Table 1 for participant characteristics). The estimated IQ of the TD group was significantly higher than that of the ADHD group *F*(1,99) = 9.31, *p* = 0.003, *η*^2^*_p_* = 0.086. Ninety-five percent of children in the TD group obtained scores on the DBDRS within the normal range for inattention and hyperactive/impulsive symptoms, i.e., between the 50th and 63.1th percentile. Children in the ADHD group all scored between the 90.9th and 97.7th percentile for inattention and hyperactive/impulsive symptoms.

### 3.2. Data Preparation and Statistical Analysis

Missing task-data occurred as a consequence of apparatus malfunction (<10%) and were missing completely at random. Data were analyzed using the statistical package SPSS version 25 [48]. Before statistical analysis, all percentage data were transformed with an arcsine transformation (preferred transformation when data is displayed in percentages [49]). Other data were subjected to the same transformation if required following review of the Skewness, Kurtosis, and Kolmogorov–Smirnov statistics, and parametric tests were conducted [49]. Due to the significant group difference for Estimated IQ and continuing debate regarding whether or not to covary for such differences in children with ADHD [50], analyses were conducted with and without IQ as a covariate. Besides IQ, age was also added as a covariate, because age can be considered a significant contributor to the outcome on a DMTS task [34].

For the CDL data, repeated measures ANOVA was conducted on the percentage of correct choices with delays as Within-Subjects Variables and group (ADHD/TD) as a Between-Subjects Factor. Group differences in visual-spatial short-term memory, visual-spatial working memory, and delay aversion were explored with a Multivariate ANOVA. To test if the relationship between ADHD and the percentage correct trials on the aDMTS (8 and 16 s) is mediated by short-term memory, working memory, or delay aversion, a multiple regression analysis with a 5000 bootstrap resamples, using a 95% confidence interval, was conducted using the process tool in SPSS [51]. To investigate the independent effects of the predictors (CDL with delays, short-term and working memory, and delay aversion) on clinical caseness (ADHD vs. TD), a binary logistic regression analysis was conducted with measures of specificity (i.e., percentage correctly classified TD children) and sensitivity (i.e., percentage correctly classified children with ADHD). Given the study design and sample size, the study had sufficient statistical power (0.80) to detect a medium effect size (*d* = 0.50) at *α* = 0.05 [52].

### 3.3. Conditional Discrimination Learning

Mauchly’s test indicated that the assumption of sphericity was violated for the main effect of delay *χ*^2^ (2) = 6.62, *p* = 0.037 of the 3 (delays) × 2 (group) repeated measures ANOVA; thus degrees of freedom were corrected using Greenhouse–Geisser. There was a significant interaction effect between delay and group in the expected direction, *F*(1.88, 185.86) = 3.52, *p* = 0.034, *ηp*^2^ = 0.034. Group comparisons, using Multivariate Analysis of Variance, indicated that the performance of children with ADHD and TD children did not differ significantly with a 0-second delay, but was significantly different at delays of 8, *F*(1, 99) = 12.94, *p* = 0.001, *ηp*^2^ = 0.116 and 16 s, *F*(1, 99) = 7.60, *p* = 0.007, *ηp*^2^ = 0.071; children with ADHD made fewer correct responses than TD children with delays of both 8 and 16 s. Controlling age and IQ strengthened this interaction effect (*p* = 0.013, *ηp*^2^ = 0.044).

**Figure 2:**
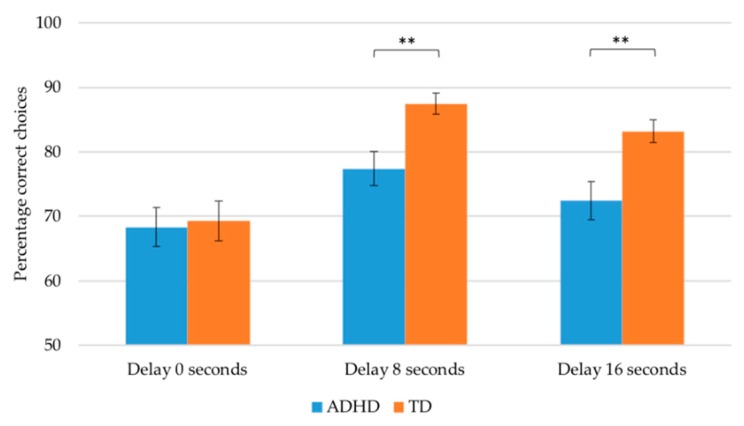
Percentage correct trials on the CDL with delays of 0, 8, and 16 s for children with ADHD (Attention Deficit Hyperactivity Disorder) and TD (Typically Developing) children. As indicated in the graph, children with ADHD performed fewer correct trials compared to TD children at delays of 8 and 16 s. ** *p* < 0.01.

In addition to the significant interaction, there was a main effect of Delay, *F*(1.88, 185.86) = 21.83, *p* < 0.001, *ηp*^2^ = 0.181 and a main effect of Group, *F*(1, 99) = 7.95, *p* = 0.006, *ηp*^2^ = 0.074. Overall, the TD children performed better compared to ADHD children, and within-group contrasts indicated that performance under each of the three delays (0, 8 and 16 s) differed significantly *F*_delay0-delay8_ (1, 99) = 41.36, *p* < 0.001, *ηp*^2^ = 0.295, *F*_delay0-delay16_ (1, 99) = 15.56, *p* < 0.001, *ηp*^2^ = 0.136, *F*_delay8-delay16_ (1, 99) = 5.94, *p* = 0.017, *ηp*^2^ = 0.057 (Figure 2). Of note, a paired-samples t-test comparing the first and last 10 trials within each delay (8 and 16 s), separately for both groups, showed a significant difference in performance during the 8-second delay for the TD children only *t*(54) = −2.07, *p* = 0.043; no other paired comparisons were significant. Both groups made the most correct choices (highest percentage) with the 8-second delay, followed by the 16 s and 0 s delay (Table 2). Both main effects remained significant after covarying for IQ and age: Delay (*p* = 0.011, *ηp*^2^ = 0.045); Group (*p* = 0.011, *ηp*^2^ = 0.065).

### 3.4. Working Memory and Delay Aversion

Results of Multivariate ANOVA showed there was a significant difference between children with ADHD and TD children on the visual-spatial short-term memory task, *F*(1, 87) = 4.62, *p* = 0.034, *ηp*^2^ = 0.050, but not the visual-spatial working memory task *F*(1, 87) = 2.50, *p* = 0.121, *ηp*^2^ = 0.027. On the delay aversion task, the MIDA, children with ADHD chose delayed reward less often, compared to the TD children, *F*(1, 87) = 4.04, *p* = 0.048, *ηp*^2^ = 0.044. When controlling for IQ and age, this effect remained significant (*p* = 0.044). The group difference for visual-spatial short-term memory was no longer significant (*p* = 0.064) following covariance analysis.

### 3.5. Mediation Model

**Figure 3:**
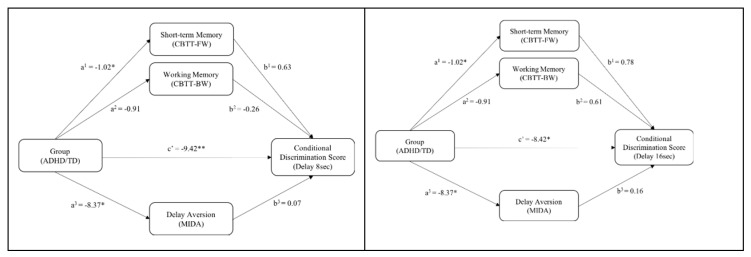
Mediation models of the outcome on an aDMTS (arbitrary Delayed Matching-To-Sample task) with 8 or 16-second delays (separately) and the mediators’ working memory, short-term memory, and delay aversion on a sample of TD and ADHD children. The presented models show that performance on the aDMTS task with delays of 8 s or 16 s was not mediated through working memory, short-term memory, or delay aversion. A clear direct negative effect between ADHD and the outcome on a CDL task with a delay (8 or 16 s) remained and increased in strength when a more dimensional ADHD-rating was used (DBDRS (Disruptive Behavior Disorder Rating Scale)-scores) or when untransformed data were analyzed. Unstandardized regression coefficients, because X is dichotomous [51]) are displayed for each relation. * *p* < 0.05. ** *p* < 0.01.

A multiple regression analysis was conducted to investigate whether having an ADHD diagnosis indirectly predicted performance on an aDMTS task through its effect on working memory, short-term memory, and delay aversion. Based on the significant differences between ADHD and TD groups, only delays of 8 and 16 s (separately) were used as outcome measures in the mediation analysis. Results showed that having ADHD did significantly predict performance on short-term memory (a^1^ = −1.02, *p* = 0.034) and delay aversion (a^3^ = −8.37, *p* = 0.048) but not on working memory. These variables, nonetheless, do not influence the performance of CDL under delays of 8 and 16 s. The bootstrap confidence intervals for all indirect effects included zero and, therefore, were not significant. Thus, clinical caseness of ADHD did not affect performance on a CDL task (8 or 16 s) through working memory, short-term memory, or delay aversion, but the effect between ADHD and CDL was direct (c’*_8sec_* = −9.42, *p* = 0.002 and c’*_16sec_* = −8.42, *p* = 0.045) (see Figure 3).

Additionally, binary logistic regression analyses were conducted to investigate the independent effects of short-term memory, working memory, delay aversion, and delay, 8 or 16 s (separately), on the group (ADHD vs. TD). When adding working memory, short-term memory, and delay aversion together with an 8-second delay in the regression, only the delay made a significant contribution to the model (B = −3.49, Wald = 8.38, *p* = 0.004, odds ratio = 0.031). The odds ratio showed that children with ADHD would have a lower percentage of correct trials with a delay of 8 s, and TD children would score higher. When using performance with a delay of 16 s, this was also the only predictor that significantly contributed to the model, (B = −2.09, Wald = 4.10, *p* = 0.043, odds ratio = 0.12). Without predictors, the overall prediction success of the model was 50.6% and increased to 68.2% with a delay of 8 s and to 62.9% with a delay of 16 s. Age and IQ were not significant predictors when added into both regression models.

## 4. Discussion

The current study is the first to experimentally evaluate daily life-choice behavior in a well-powered sample of children with and without ADHD. Choice behavior was successfully modeled using a newly developed Conditional Discrimination Learning task. The relationship between CDL performance under different delays, working memory, short-term memory, and delay aversion was assessed across the two groups using well-validated tasks. There are several noteworthy findings, the theoretical and practical relevance of which was discussed.

As predicted, children with ADHD performed more poorly than TD children on the CDL task under delay conditions, i.e., when an 8 or 16-second delay was introduced between the sample and choice stimuli. Contrary to our expectations, there was no difference in the performance of the two groups with a 0-second delay. Anticipated differences in delay aversion and short-term visual-spatial memory performance between children with ADHD and TD children were found. Children with ADHD were more delay-averse than TD children and obtained lower scores on a test of short-term memory. Performance differences in visual-spatial working memory were not significant. The hypothesis that both memory (short-term and/or working memory) and delay aversion would mediate the relationship between group membership (ADHD/TD) and CDL was not supported by mediation analyses, rather there was a direct effect of clinical caseness, i.e., between having a clinical diagnosis of ADHD and performance on the CDL task under delays of 8 and 16 s. Finally, CDL performance was the best, and only significant, predictor for classifying children with ADHD, with performance under the 8-second delay the best predictor. Together these findings suggest that a basic reinforcement learning process like CDL, *with a delay,* is impaired in ADHD. This basic learning process appears distinct from other processes, such as delay aversion and memory (short-term/working), that are implicated in ADHD.

Linking our results to daily life behavior, it would seem that children with ADHD are as able as TD children to identify or discriminate the situation/context (sample stimuli) and learn the conditions under which adaptive behavior (choice stimuli) will be reinforced when there is a limited delay (0 s) between the appearance of the context and the required behavioral response. However, when a delay is imposed, children with ADHD are less likely than TD children to perform the expected (adaptive) behavior. This is consistent with clinical reports that children with ADHD fail, or are less effective in, adapting their behavior to situational demands [53]. In the current study, delays as short as 8 s negatively impacted the performance of the children with ADHD compared to TD children. In daily life, the delay between a situational or contextual change and the required action is usually longer, e.g., a teacher announcing the topic of a class and a pupil picking the appropriate book from their bag. Under such delays, the child with ADHD may fail to perform/select the expected behavior (e.g., begin talking to their neighbor instead of retrieving a book).

The observed group differences in CDL performance might be interpreted according to theoretical constructs that emphasize the integration of contextual information as an important contributor to children’s behavioral outcome. According to Nigg and Casey [4], children with ADHD can act in line with the expectations of the environment/context once they have identified the situation. For example, having perceived their desk with a math exercise on it in the classroom, they can respond adaptively (quiet/task-oriented behavior). However, they suggest that children with ADHD may fail to recognize the full scope of the contextual information available (e.g., do not identify the math exercise) and consequently are less able to select the most adaptive response. This difficulty in integrating all the contextual information might be due to the absence of a sufficiently salient cue (e.g., a teacher giving a specific instruction to do the math task). As a result, the child does not execute the appropriate adaptive response, leading to a negative outcome (e.g., being reproved by the teacher for talking).

Extending Nigg and Casey’s theory to the current findings, the imposed delay might contribute to reducing the salience of the contextual cue. This is suggested by the current results where group differences only emerge when there is a delay between the presentation of the context (sample stimuli) and the child’s action (choice stimuli). Björne and Balkenius in their commentary on the Dynamic Developmental Theory [54], proposed that children with ADHD have a weaker memory for context, that interferes with effective conditioning of stimulus-response associations. The insertion of a delay between the presentation of the sample stimuli and the choice stimuli might further impair memory for the context in the children with ADHD in the current sample. This might also be seen as reducing the salience of a situation/context.

In addition to poorer integration of contextual cues, or memory for such information, it is important to consider other possible explanations for the current findings. The first of these relate to the symptoms of ADHD. Inattention and distractibility are core symptoms of ADHD. The introduction of delays to the CDL task may have served to reduce the attention of children with ADHD to the sample stimuli-choice stimuli association. Alternatively, having experienced a period of waiting, the children with ADHD might have responded more quickly/impulsively when the choice stimuli appeared. Failure to pay adequate attention to the sample-choice stimuli association and/or responding without careful consideration of available choices would likely reduce task performance. Although delay aversion did not mediate CDL performance, reward sensitivity might have still contributed to task performance. Tripp and Alsop proposed that children with ADHD are more influenced by the last reward received and are less able to integrate their history of reinforcement over time than TD children [55]. When delays were introduced to the CDL task, the children with ADHD might have been less able than controls to use their previous reward experiences on the task to guide their choice behavior. This could help explain the absence of a group difference with a delay of 0 s. Besides memory for the contextual cue, difficulty in using the sample stimulus-correct choice outcome to guide future behavior might also be involved.

As noted earlier, working memory performance was not significantly different between the ADHD and TD groups, nor did it mediate the relationship between group membership and CDL performance. This was somewhat surprising as differences in working memory are commonly reported in children with ADHD, although not in all studies [56], and thought to contribute to aDMTS performance. However, the skills required to perform the forward and backward CBTT (visual-spatial sequencing) might not be the same as those needed to bridge the delay between the sample and choice stimuli in a CDL paradigm. In addition to the task stimuli themselves, memory for the association between stimuli is also required in a CDL paradigm. It is possible that the children used verbal memory skills to help hold the sample stimuli and the sample-choice stimuli associations in mind. In the current study, we did not assess the children’s verbal short-term or working memory skills.

Performance on the MIDA task (delay aversion) also failed to mediate the relationship between group membership and CDL performance under conditions of delay. The lack of association between performance on the CDL task and the delay aversion task might be related to differences in the way these tasks allow the children to respond to delay. Unlike the delay aversion task, the CDL paradigm did not allow the participants to reduce the imposed delay neither by responding to reduce the waiting time nor by allowing them to engage in behaviors that would distract them from the experience of waiting. Summarizing, CDL might involve/require other different cognitive or motivational processes than those measured by the CBTT and MIDA.

As indicated in Table 2, both groups performed better at the CDL task under delay conditions than under no delay conditions. Due to the successive nature of the delays, training to task effects might have led to better performance under 8 s as compared to 0 s delay. It seems likely that during the 0 s delay, the children were still learning the task as they moved on from meaningful to non-meaningful stimuli in the practice trials. Visual inspection (Figure 2) and repeated within-group analysis (data available from the first author) also showed a similar pattern of responding over time in both the ADHD and TD groups, with differences only appearing with the addition of a delay. Also, the performance of the children with ADHD did not worsen throughout the 24 trials when delays of 8 and 16 s were introduced; thus it seems that the difference between the ADHD and TD groups cannot be attributed to progressive boredom or fatigue.

In summary, our results point towards difficulties in learning/conditioning of the adaptive behavioral response in ADHD, crucially only when a delay is present between the situational cue and the required behavior. This suggests that the capacity to adjust choice behavior to situational demands or reinforcement contingencies is a distinct process and unrelated to other pathways thought to lead or contribute to ADHD [29].

Despite the many strengths of the current study, including sample size, group assignment, and the CDL paradigm itself, some limitations need to be acknowledged that reduce the interpretations that can be made. In relation to the sample itself, symptom severity in the ADHD group may be moderate only. In addition to reducing the generalizability of the findings to more impaired samples, this may have restricted the range of scores on the predictor variables, making it more difficult to demonstrate mediation. While the sample size is good, the distribution of ADHD subtypes did not permit us to assess mediation effects in more homogenous subsamples. While care was taken in piloting the task to establish the appropriate difficulty level, additional practice trials might be needed ahead of testing to ensure participants are comfortable with the task.

## 5. Clinical Implications

The findings of the current study have important implications for managing and/or accommodating the behavioral difficulties of children with ADHD. The adaptive choice behavior of children with ADHD does not differ from that of TD children when contextual cues are salient/in close temporal proximity to the behavioral choice/response. When delays are introduced, children with ADHD are less able than TD children to use contextual cues to guide their behavior. The precise mechanisms underlying this reduced efficiency of situational cues/context are uncertain, but the findings are clear.

For children with ADHD, it will be important to reduce delays between the introduction of a new situation/context and requiring the child to respond. This is not always possible in daily life; however, the impact of a delay may be lessened by making the context more salient for the child through the use of stimulus control techniques. According to nigg and casey [4], children with ADHD know what the most adaptive response is once they realize the context they are currently in; however, they are not able to take on board all the contextual cues to do so. Presenting information through multiple modalities is one possible approach, e.g., showing as well as telling the child with ADHD what is required (e.g., class rules on a chart or near their desk) may increase the salience of the context and remind the child to use that information, especially in situations of delay. Caregivers can also support children with ADHD to attend to relevant situational cues, encouraging them to select the best response based on all the information available to them (e.g. “in the classroom, I should be sitting at my desk and not running around”).

Another intervention that may enhance CDL under conditions of delay is a Differential Outcomes procedure (DO) [12,57,58]. In a DO procedure, the association between the sample and choice stimuli is strengthened by the addition of a unique outcome rather than a generic one. This procedure is often used in clinical samples with memory impairments (e.g., Prader–Willi, dementia, Korsakoff). By changing the associative structure, more conditioning can take place, overcoming the delay. In DO, correctly choosing C_1_ after the presentation of S_1_ will result in outcome O_1_, while correctly choosing C_2_ after the presentation of S_2_ will result in a different outcome O_2_ [58,59]. In DO, sample stimuli come to elicit specific outcome anticipations, thereby increasing CDL. Although not yet tested in ADHD, DO procedures are effective in improving CDL in Prader–Willi and Korsakoff syndromes [58].

## Figures and Tables

**Table 1:** Demographic and Clinical Characteristics of Children in the ADHD and TD Groups.

	**ADHD**	**TD**	***F/χ*** ^**2**^	***p***
	**M (SD)**	**M (SD)**		
*Gender N*			3.43	0.064
Male	31	27		
Female	15	28		
Age (years)	10.28 (0.98)	10.01 (1.21)	1.46	0.230
FSIQ	98.04 (11.60)	104.71 (10.35)	9.31	0.003 **
Dyscalculia (%)	2.17	0	1.21	0.272
Dyslexia (%)	8.70	0	4.98	0.026 *
*DBDRS*				
Attention	14.95 (1.79)	10.52 (0.99)	241.84	<0.001 ***
Hyperactive/impulsive	14.36 (2.17)	10.54 (0.97)	135.46	<0.001 ***
ODD	12.55 (2.13)	10.72 (1.24)	28.06	<0.001 ***
CD	11.45 (1.53)	10.93 (1.28)	3.86	0.052

Note. ADHD = Attention Deficit Hyperactivity Disorder; TD = Typically Developing; FSIQ: Full-Scale IQ; DBDRS = Disruptive Behavior Rating Scale; ODD = Oppositional Defiant Disorder; CD = Conduct Disorder. * *p* < 0.05, ** *p* < 0.01, *** *p* < 0.001.

**Table 2:** Untransformed Mean Percentages, Standard Deviations, and Multivariate ANOVA Results for the Conditional Discrimination Learning, Short-Term Memory, Working Memory, and Delay Aversion tasks: ADHD and Control Groups.

	ADHD	TD			
	M (SD)	M (SD)	*F* ^1^	*p* ^1^	*η* ^2^ *_p_*
Delay 0	68.34 (20.68)	69.26 (23.10)	0.14	0.711	0.001
Delay 8	77.39 (17.76)	87.45 (12.13)	12.94	0.001 **	0.116
Delay 16	72.39 (20.10)	83.18 (13.07)	7.60	0.007 **	0.071
Short-term Memory ^2^	42.47 (13.62)	48.11 (11.24)	4.62	0.034 *	0.050
Working Memory ^2^	43.58 (13.81)	48.61 (15.63)	2.50	0.121	0.027
Delay Aversion ^2^	81.39 (19.75)	89.76 (14.30)	4.04	0.048 *	0.044

ADHD = Attention Deficit Hyperactivity Disorder; TD = Typically Developing. * *p* < 0.05, ** *p* < 0.01. ^1^ Based on transformed data. ^2^ Due to missing data (computer/task failure n = 12), Short-Term Memory, Working Memory and Delay Aversion analysis and means are based on the participants for whom all of the data were available (44 TD children and 45 ADHD children). The means for the whole data set are available from the first author.

## References

[1] Mok L.W., Estevez A.F., Overmier J.B. (2017). Unique Outcome Expectations as a Training and Pedagogical Tool. Psychol. Rec..

[2] Schultz W. (2000). Multiple reward signals in the brain. Nat. Rev. Neurosci..

[3] Alsop B., Furukawa E., Sowerby P., Jensen S., Moffat C., Tripp G. (2016). Behavioral sensitivity to changing reinforcement contingencies in attention-deficit hyperactivity disorder. J. Child Psychol. Psychiatry Allied Discip..

[4] Nigg J.T., Casey B.J. (2005). An integrative theory of attention-deficit/hyperactivity disorder based on the cognitive and affective neurosciences. Dev. Psychopathol..

[5] De Meyer H., Beckers T., Tripp G., van der Oord S. (2019). Reinforcement Contingency Learning in Children with ADHD: Back to the Basics of Behavior Therapy. J. Abnorm. Child Psychol..

[6] Cunningham J.S., Knights R.M. (1978). The performance of hyperactive and normal boys under differing reward and punishment schedules. J. Pediatr. Psychol..

[7] Douglas V.I., Parry P.A., Parrry P.A. (1983). Effects of reward on delayed reaction time task performance of hyperactive children. J. Abnorm. Child Psychol..

[8] Douglas V.I., Parry P.A. (1994). Effects of reward and nonreward on frustration and attention in attention deficit disorder. J. Abnorm. Child Psychol..

[9] Freibergs V., Douglas V.I. (1969). Concept learning in hyperactive and normal children. J. Abnorm. Psychol..

[10] Segers E., Beckers T., Geurts H., Claes L., Danckaerts M., van der Oord S. (2018). Working memory and reinforcement schedule jointly determine reinforcement learning in children: Potential implications for behavioral parent training. Front. Psychol..

[11] Trapold M.A. (1970). Are expectancies based upon different positive reinforcing events discriminably different?. Learn. Motiv..

[12] Estévez A.F., Fuentes L.J., Marí-Beffa P., González C., Alvarez D. (2001). The Differential Outcome Effect as a Useful Tool to Improve Conditional Discrimination Learning in Children. Learn. Motiv..

[13] Frank M.J., Scheres A., Sherman S.J. (2011). Understanding decision-making deficits in neurological conditions: Insights from models of natural action selection. Model. Nat. Action Sel..

[14] Gitten J.C., Winer J.L., Festa E.K., Heindel W.C. (2006). Conditional associative learning of spatial and object information in children with attention deficit/hyperactivity disorder. Child Neuropsychol..

[15] Sonuga-Barke E.J.S. (2003). The dual pathway model of AD/HD: An elaboration of neuro-developmental characteristics. Neurosci. Biobehav. Rev..

[16] Wehmeier P.M., Schacht A., Barkley R.A. (2010). Social and Emotional Impairment in Children and Adolescents with ADHD and the Impact on Quality of Life. J. Adolesc. Health.

[17] Biederman J., Monuteaux M.C., Doyle A.E., Seidman L.J., Wilens T.E., Ferrero F., Morgan C.L., Faraone S.V. (2004). Impact of executive function deficits and attention-deficit/hyperactivity disorder (ADHD) on academic outcomes in children. J. Consult. Clin. Psychol..

[18] Sonuga-Barke E.J.S., Sergeant J.A., Nigg J., Willcutt E. (2008). Executive Dysfunction and Delay Aversion in Attention Deficit Hyperactivity Disorder: Nosologic and Diagnostic Implications. Child Adolesc. Psychiatr. Clin. N. Am..

[19] Martinussen R., Hayden J., Hogg-Johnson S., Tannock R. (2005). A meta-analysis of working memory impairments in children with attention-deficit/hyperactivity disorder. J. Am. Acad. Child Adolesc. Psychiatry.

[20] Kofler M.J., Harmon S.L., Aduen P.A., Day T.N., Austin K.E., Spiegel J.A., Irwin L., Sarver D.E. (2018). Neurocognitive and behavioral predictors of social problems in ADHD: A bayesian framework. Neuropsychology.

[21] Willcutt E.G., Doyle A.E., Nigg J.T., Faraone S.V., Pennington B.F. (2005). Validity of the Executive Function Theory of Attention-Deficit/Hyperactivity Disorder: A Meta-Analytic Review. Biol. Psychiatry.

[22] Dovis S., Van Der Oord S., Wiers R.W., Prins P.J.M. (2013). What part of working memory is not working in ADHD? short-term memory, the central executive and effects of reinforcement. J. Abnorm. Child Psychol..

[23] Conway A.R.A., Cowan N., Bunting M.F. (2001). The cocktail party phenomenon revisited: The importance of working memory capacity. Psychon. Bull. Rev..

[24] Luman M., Tripp G., Scheres A. (2010). Identifying the neurobiology of altered reinforcement sensitivity in ADHD: A review and research agenda. Neurosci. Biobehav. Rev..

[25] Sagvolden T., Johansen E.B., Aase H., Russell V.A. (2005). A dynamic developmental theory of attention-deficit/hyperactivity disorder (ADHD) predominantly hyperactive/impulsive and combined subtypes. Behav. Brain Sci..

[26] Tripp G., Wickens J.R. (2008). Research review: Dopamine transfer deficit: A neurobiological theory of altered reinforcement mechanisms in ADHD. J. Child Psychol. Psychiatry Allied Discip..

[27] Van Dessel J., Sonuga-Barke E., Mies G., Lemiere J., Van der Oord S., Morsink S., Danckaerts M. (2018). Delay aversion in attention deficit/hyperactivity disorder is mediated by amygdala and prefrontal cortex hyper-activation. J. Child Psychol. Psychiatry Allied Discip..

[28] Bitsakou P., Antrop I., Wiersema J.R., Sonuga-Barke E.J.S. (2006). Probing the limits of delay intolerance: Preliminary young adult data from the Delay Frustration Task (DeFT). J. Neurosci. Methods.

[29] Coghill D., Seth S., Matthews K. (2014). A comprehensive assessment of memory, delay aversion, timing, inhibition, decision making and variability in attention deficit hyperactivity disorder: Advancing beyond the three-pathway models. Psychol. Med..

[30] Frank M.J., Santamaria A., Reilly R.C., Willcutt E., O’Reilly R.C., Willcutt E. (2007). Testing computational models of dopamine and noradrenaline dysfunction in attention deficit/hyperactivity disorder. Neuropsychopharmacology.

[31] Adamson C., Foster T.M., McEwan J.S.A. (2000). Delayed matching to sample: The effects of sample-set size on human performance. Behav. Processes.

[32] Grant R.A. (1976). The relation of perceptual activity to Matching Familiar Figures Test accuracy. Dev. Psychol..

[33] Etkin M., D’Amato M.R. (1969). Delayed matching-to-sample and short-term memory in the capuchin monkey. J. Comp. Physiol. Psychol..

[34] Chelonis J.J., Daniels-Shaw J.L., Blake D.J., Paule M.G. (2000). Developmental aspects of delayed matching-to-sample task performance in children. Neurotoxicol. Teratol..

[35] Elliott R., Dolan R.J. (1999). Differential neural responses during performance of matching and nonmatching to sample tasks at two delay intervals. J. Neurosci..

[36] Case J.P., Laude J.R., Zentall T.R. (2015). Delayed matching to sample in pigeons: Effects of delay of reinforcement and illuminated delays. Learn. Motiv..

[37] Skinner B.F. (1950). Are theories of learning necessary?. Psychol. Rev..

[38] Shaffer D., Fisher P., Lucas C.P., Dulcan M.K., Schwab-Stone M.E. (2000). NIMH Diagnostic Interview Schedule for Children Version IV (NIMH DISC-IV): Description, Differences From Previous Versions, and Reliability of Some Common Diagnoses. J. Am. Acad. Child Adolesc. Psychiatry.

[39] Kort W., Schittekatte M., Bosmans M., Compaan E.L., Dekker P.H., Vermeir G., Verhaeghe P. (2005). WISC-III: Handleiding En Verantwoording.

[40] Sattler J. (2001). Assessment of Children: Cognitive Applications.

[41] Oosterlaan J., Baeyens D., Scheres A., Antrop I., Roeyens H., Sergeant J. (2008). VvGK6-16: Vragenlijst Voor Gedragsproblemen Bij Kinderen 6 Tot En Met 16 Jaar.

[42] Greenhill L.L., Nathan P.E., Gorman J. (1998). A guide to treatments that work. A Guide to Treatments That Work.

[43] Domjan M. (2000). The Essentials of Conditioning and Learning.

[44] Corsi P.M. (1972). Human memory and the medial temporal region of the brain. Diss. Abstr. Int..

[45] Kuntsi J., Stevenson J., Oosterlaan J., Sonuga-Barke E.J.S. (2001). Test-retest reliability of a new delay aversion task and executive function measures. Br. J. Dev. Psychol..

[46] Kuntsi J., Oosterlaan J., Stevenson J. (2001). Psychological Mechanisms in Hyperactivity: I Response Inhibition Deficit, Working Memory Impairment, Delay Aversion, or Something Else?. J. Child Psychol. Psychiatry.

[47] American Psychiatric Association (2000). Diagnostic and Statistical Manual of Mental Disorders: DSM-IV-TR.

[48] IBM Corp (2017). IBM SPSS Statistics for Macintosh, Version 25.0.

[49] Zar J.H., Englewood Cliffs N.J. (1984). Biostatistical Analysis.

[50] Dennis M., Francis D.J., Cirino P.T., Schachar R., Barnes M.A., Fletcher J.M. (2009). Why IQ is not a covariate in cognitive studies of neurodevelopmental disorders. J. Int. Neuropsychol. Soc..

[51] Hayes A.F. (2017). Introduction to Mediation, Moderation, and Conditional Process Analysis.

[52] Faul F., Erdfelder E., Lang A.-G., Buchner A. (2007). G*Power 3: A flexible statistical power analysis program for the social, behavioral, and biomedical sciences. Behav. Res. Methods.

[53] American Psychiatric Association (2013). Diagnostic and Statistical Manual of Mental Disorders.

[54] Björne P., Balkenius C. (2005). The role of context and inhibition in ADHD. Behav Brain Sci..

[55] Tripp G., Alsop B. (1999). Sensitivity to Reward Frequency in Boys with Attention Deficit Hyperactivity Disorder. J. Clin. Child Adolesc. Psychol..

[56] Geurts H.M., Verté S., Oosterlaan J., Roeyers H., Sergeant J.A. (2004). How specific are executive functioning deficits in attention deficit hyperactivity disorders and autism?. J. Child Psychol. Psychiatry Allied Discip..

[57] Mok L.W., Overmier J.B. (2007). The differential outcomes effect in normal human adults using a concurrent-task within-subjects design and sensory outcomes. Psychol. Rec..

[58] Overmier J.B., Linwick D. (2001). Conditional Choice-Unique Outcomes Establish Expectancies That Mediate Choice Behavior. Integr. Physiol. Behav. Sci..

[59] Holden J.M., Overmier J.B. (2014). Performance under differential outcomes: Contributions of Reward-Specific Expectancies. Learn. Motiv..

